# Downsizing of rectal cancer following neoadjuvant radiotherapy (5 × 5 Gy) and long interval surgery evaluated using MRI semiautomated volumetric measurements, a retrospective study

**DOI:** 10.3389/fsurg.2023.1106177

**Published:** 2023-02-17

**Authors:** Hendrik Christian Albrecht, Sophie Wagner, Christoph Sandbrink, Stephan Gretschel

**Affiliations:** ^1^ Department of General, Visceral, Thoracic and Vascular Surgery, University Hospital Ruppin- Brandenburg, Neuruppin, Germany; ^2^ Faculty of Health Sciences Brandenburg, Brandenburg Medical School Theodor Fontane, Neuruppin, Germany

**Keywords:** total tumor volume measurement, short-term radiation with delayed surgery, tumor downsizing, neoadjuvant therapy, rectal cancer

## Abstract

**Introduction:**

Neoadjuvant conventional chemoradiation (CRT) is the standard treatment for primary locally non-curatively resectable rectal cancer, as tumor downsizing may allow R0 resectability. Short-term neoadjuvant radiotherapy (5x5 Gy) followed by an interval before surgery (SRT- delay) is an alternative for multimorbid patients who cannot tolerate CRT. This study examined the extent of tumor downsizing achieved with the SRT-delay approach in a limited cohort that underwent complete re-staging before surgery.

**Methods:**

Between March 2018 and July 2021, 26 patients with locally advanced primary adenocarcinoma (>uT3 or/and N+) of the rectum were treated with SRT-delay. 22 patients underwent initial staging and complete re-staging (CT, endoscopy, MRI). Tumor downsizing was assessed by staging and re-staging data and pathologic findings. Semiautomated measurement of tumor volume was performed using mint Lesion™ 1.8 software to evaluate tumor regression.

**Results:**

The mean tumor diameter determined on sagittal T2 MRI images decreased significantly from 54.1 (23–78) mm at initial staging to 37.9 (18–65) mm at re-staging before surgery (p <0.001) and to 25.5 (7–58) mm at pathologic examination (p <0.001). This corresponds to a mean reduction in tumor diameter of 28.9 (4.3–60.7) % at re-staging and 51.1 (8.7–86.5) % at pathology. Mean tumor volume determined from transverse T2 MR images mint Lesion^TM^ 1.8 software significantly decreased from 27.5 (9.8 – 89.6) cm^3^ at initial staging to 13.1 (3.7 – 32.8) cm^3^ at re-staging (p <0.001), corresponding to a mean reduction of 50.8 (21.6 – 77) %. The frequency of positive circumferential resection margin (CRM) (less than 1mm) decreased from 45,5 % (10 patients) at initial staging to 18,2 % (4 patients) at re-staging. On pathologic examination, the CRM was negative in all cases. However, multivisceral resection for T4 tumors was required in 2 patients (9%). Tumor downstaging was noted in 15 of 22 patients after SRT-delay.

**Conclusion:**

In conclusion, the observed extent of downsizing is broadly comparable to the results of CRT, making SRT-delay a serious alternative for patients who cannot tolerate chemotherapy.

## Introduction

Colorectal carcinoma is one of the most common malignant tumors of the digestive tract and a relevant cause of cancer-related deaths. It is the third most common tumor disease in both sexes worldwide and the second leading cause of death among all cancers (1).

In rectal cancer, local recurrence is an important problem that affects not only oncologic outcomes but also quality of life.

The establishment of the concept of total mesorectal excision (TME) as a standard procedure (2) and the introduction of neoadjuvant therapy for locally advanced tumors have contributed to the improvement of local control in rectal cancer in recent decades (3–5).

In terms of oncologic outcome in locally advanced, resectable rectal cancer, short-term neoadjuvant irradiation (5 × 5 Gy) (SRT) and surgery the following week were shown to be equivalent to long-term neoadjuvant chemoradiation (28 × 1.8 Gy, 5-fluorouracil, and leucovorin) and surgery after 4–6 weeks (CRT) (6).

For primary locally non-curatively resectable tumors with infiltration of the pelvic wall or floor, adjacent organs, or sphincter, conventional long-term neoadjuvant chemoradiation remains the standard of care, as tumor downsizing may allow R0 resectability or sphincter-preserving resection (7, 8). Recently, even more aggressive concepts of total neoadjuvant therapy have been introduced, achieving complete remission in up to 30% of cases, even in extensive tumors (9–11).

However, a proportion of elderly multimorbid patients do not tolerate even standard long-term neoadjuvant chemoradiation. Therefore, a concept of short-term neoadjuvant radiotherapy (5 × 5 Gy) followed by a 4–8-week interval before surgery (SRT-delay), with the goal of tumor regression, was developed for these patients.

The feasibility of the SRT-delay approach has already been demonstrated in studies without evidence of increased complication rates (12–14).

The extent of downsizing achieved with this approach has not yet been systematically studied.

The few reports of tumor regression with SRT-delay are mainly based on pathologic findings compared with initial clinical and radiologic staging. To date, there are no tumor downsizing data with this neoadjuvant approach in the context of re-staging data, particularly no data measuring total tumor volume.

The aim of this study was to evaluate the extend of downsizing of locally advanced rectal cancer in the SRT-delay approach in a limited cohort undergoing complete re-staging in the interval before surgery. In addition, we aimed to investigate the total tumor volume to assess the downsizing of rectal cancer after this neoadjuvant approach.

## Patients and methods

### Patients

Between March 2018 and July 2021, 26 patients were treated with the concept of neoadjuvant radiotherapy (5 × 5 Gy) and delayed surgery (SRT-delay) for rectal cancer at Ruppin- Brandenburg University Hospital. All patients had locally advanced primary adenocarcinoma (≥uT3 or/and N+) in the lower or middle third of the rectum. In addition, patients with locally advanced upper third rectal cancer whose main tumor mass appeared caudal to the promontory on sagittal MRI view were included.

Patients were assigned to this form of neoadjuvant therapy because they either could not tolerate conventional neoadjuvant chemoradiation due to their comorbidities or refused chemotherapy.

Short-term neoadjuvant radiotherapy included five fractions of 5 Gy in one week (5 × 5 Gy), followed by an interval of about 8 weeks before surgery.

22 of the 26 patients underwent initial staging (CT, endoscopy, MRI) and complete re-staging before surgery. 4 of 26 patients had to be excluded from the study because of insufficient re-staging. In 2 of these 4 cases, inserted hip arthroplasties caused poor MRI quality. MRI was not possible in one patient, and re-staging endoscopy was not performed in the remaining patient.

The clinical data of the 22 patients enrolled in the study are shown in Table 1.

**Table 1:** Clinical data of patients included. mrCRM, circumferential resection margin at primary staging MRI.

Age (years)	72.3 (mean)	51–84 (range)
	Male	Female
**Sex (%)**	68.2	31.8
**SRT-delay (weeks)**	8.1 (mean)	4.3–10.9 (range)
**UICC Stage (%)**	**I**	1 (4.5)
	**II**	6 (27.3)
	**III**	14 (63.6)
	**IV**	1 (4.5)
**Primary T staging**	**2**	1
	**3a**	3
	**3b**	3
	**3c**	12
	**3d**	2
	**4a**	0
	**4b**	1
**Primary N staging**	0	7
	1	14
	2	1
**Tumor height**		
**Low (<6 cm)**	4	
**Mid (6–12 cm)**	14	
**High (>12 cm)**	4	
	**<1 mm**	**>1 mm**
**mrCRM (%)**	45.5	54.5

The study was approved by the ethics committee of the Brandenburg Medical School (No. E-02-20210930).

Tumor downsizing after neoadjuvant therapy was assessed by comparing staging and re-staging data on MRI and endoscopy and by comparing initial staging data with pathological findings.

### MRI

The largest tumor diameter was determined using pelvic MRI in mercury technique in sagittal T2 images as crania-caudal extension. Tumor diameters were defined as D1 at initial staging and D2 at re-staging before surgery.

The distance of the tumor to the mesorectal fascia (MRF) was assessed in the transversal T2 images and classified as greater or less than 1 mm.

In addition, tumor volume was determined at initial staging (V1) and re-staging (V2).

**Figure 1:**
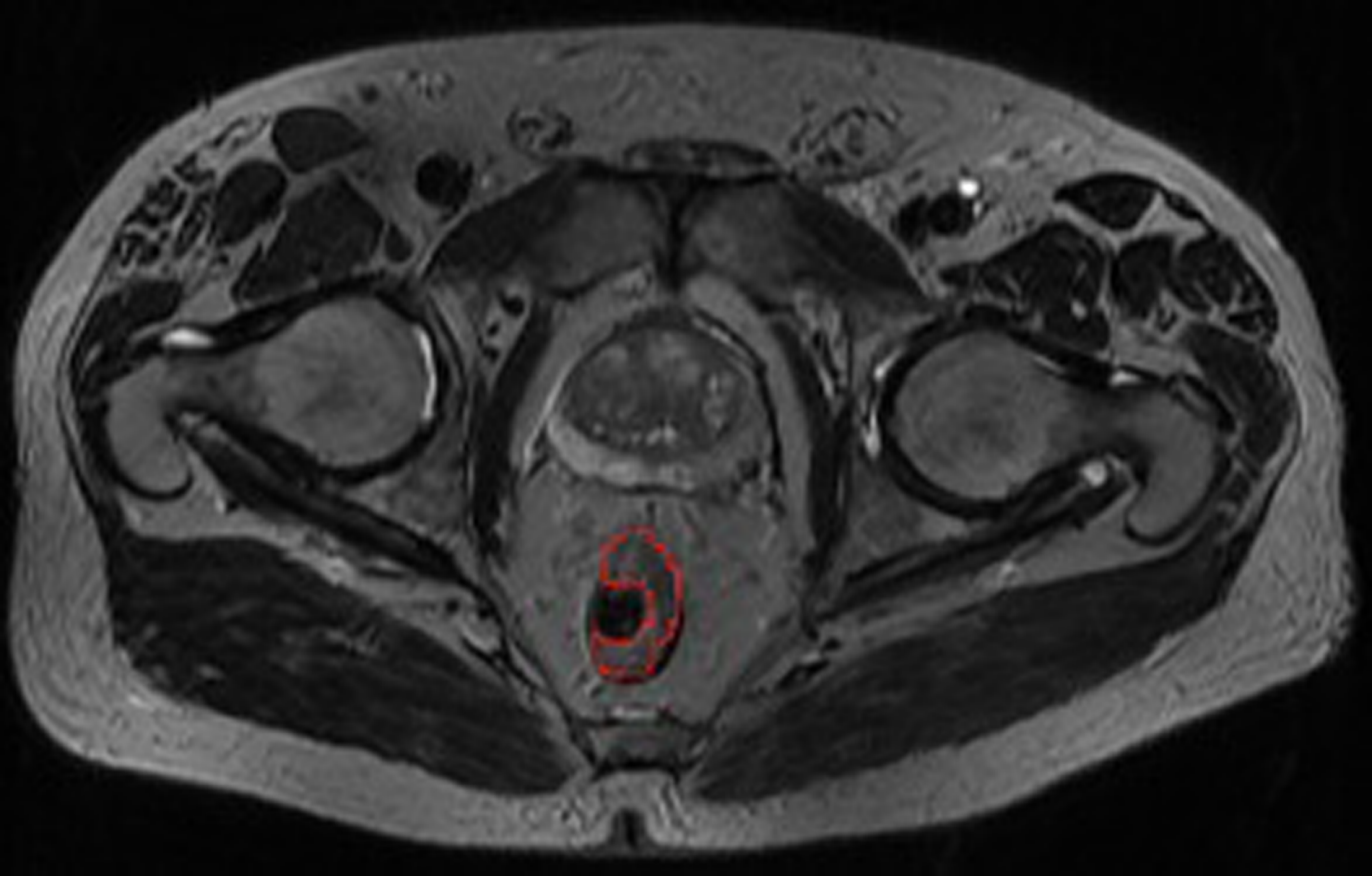
Entire tumor margins marked (red)—one of three marked transverse images for semiautomated volume measurement using mint lesion^TM^ 1.8.

For semiautomated volume measurement, the entire tumor margins were marked by an experienced radiologist using mint Lesion^TM^ 1.8 radiology software (Mint Medical, Dossenheim, Germany) on three transverse T2 images of the tumor: the most cranial, the most caudal, and one additional (Figure 1).

The mint Lesion^TM^ software interpolated the tumor margins in the remaining, non-manually marked transverse T2 images and calculated the corresponding volume. In case of differences between the interpolated margins and the actual tumor margins, the interpolated margins were manually corrected.

MRI images were evaluated by two experienced radiologists who independently assessed tumor diameter and total tumor volume. The mean value of both examiners was used for further analysis.

### Endoscopy

Rigid and flexible rectoscopy were used at baseline and re-staging to assess the tumor and the distance of the aboral tumor margin from the anal verge.

For semiquantitative assessment of tumor downsizing after neoadjuvant therapy, the endoscopy was performed by the same investigator.

The endoscopist evaluated tumor changes comparing staging and re-staging using the following classification:

0 - no changes/progression
1 - moderate regression up to 25%.
2 - significant regression 25–75%
3 - extensive regression > 75%
4 - not assessable

Category 4 concerned stenosing tumors that were also stenosing at re-staging. Possible changes could not be assessed endoscopically in these cases.

### Pathological examination

Circumferential resection margin (CRM) was defined as negative if the distance of the tumor from the margin was more than 1 mm. Histopathological tumor regression to neoadjuvant radiotherapy was evaluated according to the Dworak scoring system (15). The quality of the TME was evaluated using the protocol introduced by Quirke (16).

### Statistical evaluation

Statistical analysis was performed using GraphPad Prism 9 (GraphPad Software, LLC, San Diego, CA). Descriptive statistics in the form of mean and standard deviation were obtained and presented as tables and box plots. Changes in tumor size etc. were analyzed using the paired t test. When more than two groups were compared, a one-way ANOVA was performed with Tukey's multiple comparisons test. Interobserver correlations were calculated using the Pearson correlation coefficient. The overall significance level was set at *α* = 0.05 and marked with an * in the graphs. A significance level of *α* = 0.01 was marked with ** and *α* = 0.001 with ***.

### Patients follow-up

Patient follow-up was scheduled according to the German guideline for colorectal cancer. Follow up included a medical history and physical examination, blood tests such as serum carcinoembryonic antigen (CEA), sonography, rectoscopy every 6 months. Computed tomography of the chest, abdomen, and pelvis and colonoscopy were performed annually. Patients who did not show up for examinations were followed up by telephone.

## Results

Of the 22 patients enrolled in the study, 7 were women and 15 were men. The mean age was 72 (51–84) years. The interval between radiotherapy and surgery averaged 8.1 (4.3–10.9) weeks. Oncologic (low) anterior resection of the rectum with total mesorectal excision and central lymphadenectomy (low tie of inferior mesenteric artery) was performed in 18 patients. One patient underwent intersphincteric resection followed by hand-sewn coloanal anastomosis.

Multivisceral resection was required in 3 patients, *en bloc* hysterectomy in one patient, and *en bloc* resection of the urinary bladder in another. The third patient underwent extended abdomino-perineal excision with *en bloc* partial vaginectomy. Fifteen patients underwent laparoscopic surgery, and 7 patients had open surgery.

### MRI

**Figure 2:**
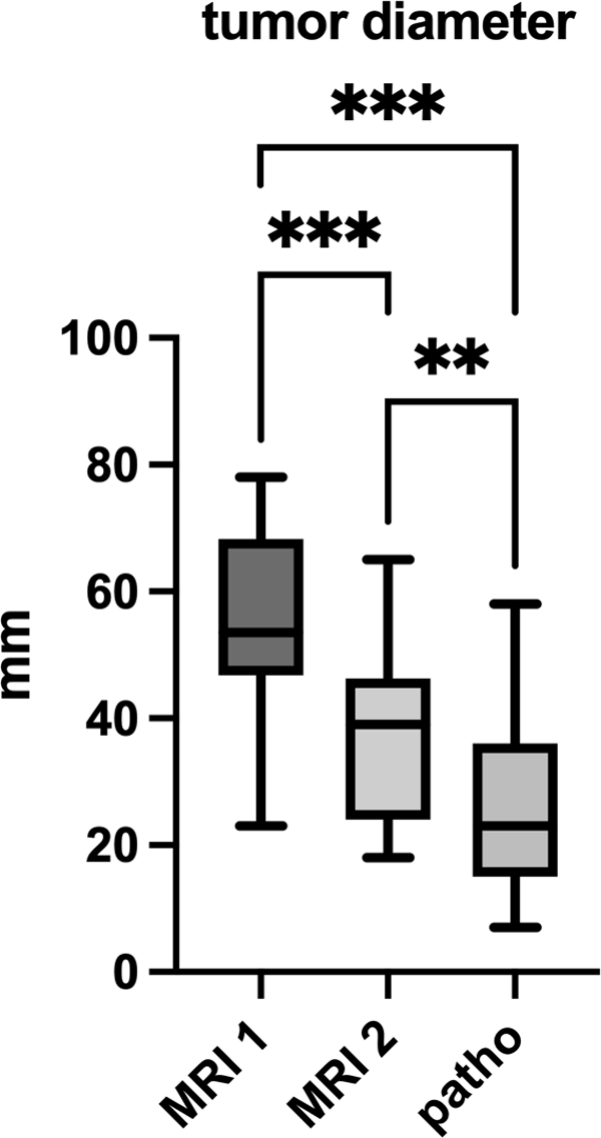
Tumor diameter in mm in staging MRI 1, MRI 2 and pathological examination. ** significance level *α* = 0.01, *** *α* = 0.001.

The mean value of the largest tumor diameter, determined as cranio-caudal extent in sagittal T2 images, decreased significantly from 54.1 (23–78) mm at initial staging (D1) to 37.9 (18–65) mm at re-staging before surgery (D2) (*p* < 0.001) (Figure 2). This corresponds to a mean reduction in cranio-caudal tumor diameter of 28.9 (4.3–60.7) %.

Evaluation of the distance of the tumor from the mesorectal fascia in transverse T2 images showed that it was less than 1 mm in 10 patients (45.5%) at initial staging, but only in 4 patients (18.2%) at re-staging.

Metastatic lymph node involvement was detected in 15 patients at initial staging and in 7 patients at re-staging.

**Figure 3:**
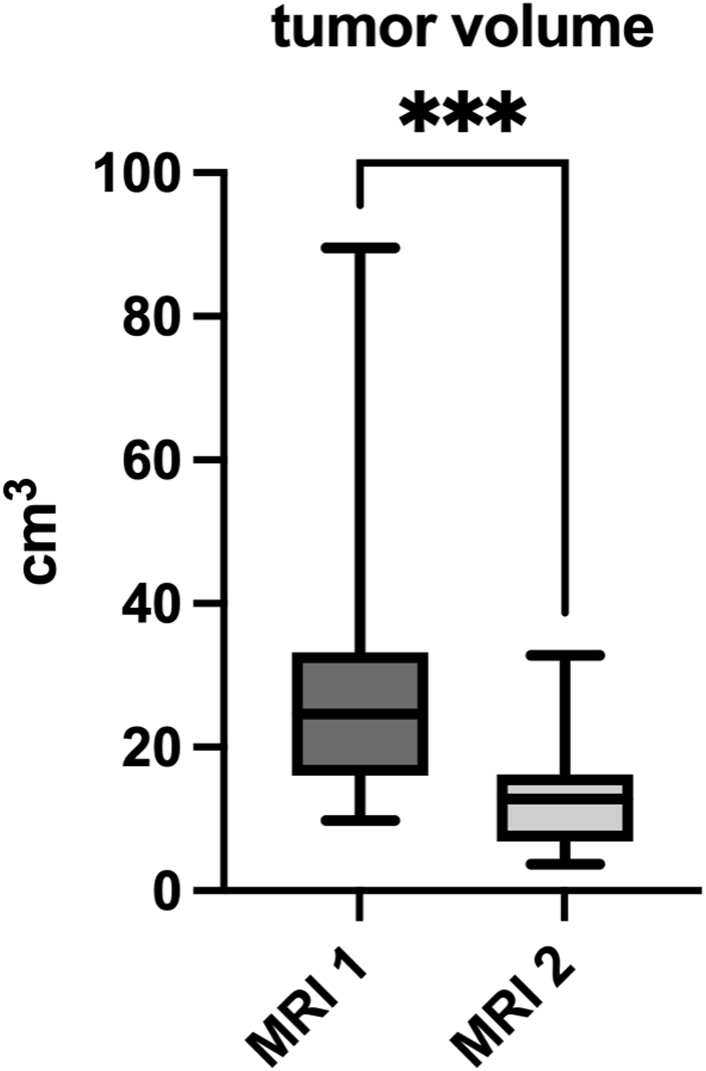
Tumor volume in cm3 measured in primary- and re-staging MRI. *** significance level *α* = 0.001.

Semiautomated volume measurement using mint Lesion^TM^ 1.8 software revealed a significant decrease in mean tumor volume from 27.5 (9.8–89.6) cm^3^ at initial staging to 13.1 (3.7–32.8) cm^3^ at re-staging (*p* < 0.001) (Figure 3). This equates to a mean reduction in tumor volume after neoadjuvant radiotherapy of 50.8 (21.6–77) %.

Analysis of interobserver reliability revealed a Pearson correlation coefficient of r = 0.95 for tumor diameter D1 on MRI 1 and r = 0.97 for D2 on MRI 2. Regarding tumor volume, the Pearson correlation coefficient was r = 0.99 for V1 in MRI 1 and r = 0.97 for V2 in MRI 2.

### Endoscopy

Semiquantitative endoscopic assessment of the tumor after neoadjuvant therapy revealed moderate regression (up to 25%) in 5 patients, significant regression (25%–50%) in 8 patients, and extensive regression (>75%) in another 5 patients.

Endoscopic assessment of tumor changes after neoadjuvant therapy could not be performed in 4 patients because the tumors were stenosing at both initial and re-staging.

### Pathological examination

R0 resection of the tumor was achieved in all 22 patients. The circumferential resection margin was negative in all cases and not smaller than 1 mm.

The mean tumor size at pathological examination was 25.5 (7–58) mm (Figure 2).

This corresponds to a significant reduction in mean tumor diameter compared to initial staging MRI (D1) (*p* < 0.001) of 51.1 (8.7–86.5) % on average.

Lymph node metastases were found in the specimens of 6 patients.

Tumor regression according to the Dworak classification was grade 1 in 10 patients, grade 2 in 8 patients, and grade 3 in 3 patients. Only in one patient, no histopathological tumor regression could be observed after neoadjuvant radiation (Dworak grade 0).

**Figure 4:**
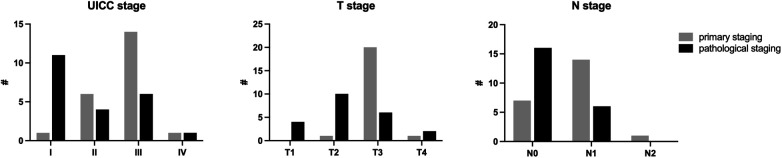
UICC, T and N stage in primary staging and pathological findings.

At pathological staging, tumor downstaging was noted in 15 of 22 patients after neoadjuvant therapy compared with initial staging (Figure 4).

### Follow up

After a mean follow-up of 34.6 (14–54) months, 3 of 26 patients had died unrelated to tumor. Another patient had died from multiple distant metastases. 3 other patients developed distant metastases, 2 of whom had solitary metastases that were surgically resected. One patient had a recurrence of rectal cancer. On pathologic examination, the tumor was found to have grown from an HGIEN polyp. Therefore, it was considered a *de novo* metachronous second rectal cancer rather than a local recurrence. In 18 of 26 patients, there was no evidence of new tumor manifestations. Of the above patients, 2 did not show up for scheduled examinations and were therefore followed up by telephone.

In summary, with a mean follow-up of 34.6 (14–54) months, disease free survival was seen in 18 of 26 patients (69,2%) and overall survival in 22 of 26 patients (84,6%).

## Discussion

Neoadjuvant radiotherapy has become the standard of care for locally advanced rectal cancer, as both hyperfractionated radiotherapy and conventional chemoradiation (CRT) have been shown to reduce the rate of local recurrence (3–6). These results are also consistent in the cohort of patients treated with the surgical standard of TME (4, 17). The most important risk factors for locoregional recurrence are involvement of the circumferential resection margin and positive lymph node status (5, 18). The quality of surgery (controlled TME, number of lymph nodes retrieved) influences the latter. However, in noncuratively resectable tumors with infiltration of the mesorectal fascia, pelvic wall or floor only downsizing following neoadjuvant therapy may enable resection with a sufficiently wide negative (>1 mm) circumferential margin.

From this perspective, evaluation of the chances of short-term neoadjuvant radiotherapy (5 × 5 Gy) with delayed surgery (SRT-delay) as an alternative to conventional chemotherapy in patients who cannot tolerate chemotherapy depends on the extent of tumor downsizing achieved with this approach. In addition, the subgroup of patients responding to neoadjuvant therapy with tumor downstaging was shown to have a survival benefit (14, 19, 20).

For SRT-delay, reports of tumor regression are mainly based on pathologic findings compared with initial clinical and radiologic staging (12–14). However, MRI is known to have limitations in predicting tumor and lymph node category with a tendency to overstaging. On the other hand, prediction of mesorectal fascia involvement and positive CRM by MRI is considered very accurate (21).

In our study, the frequency of positive CRM decreased from 45% (10 patients) at initial staging to 18% (4 patients) at re-staging. On pathologic examination, the circumferential resection margin was negative in all cases. However, in 3 patients (14%) with T4 tumors requiring multivisceral *en bloc* resection for negative CRM, the mesorectal fascia (MRF) remained infiltrated. This issue of correct terminology in initial staging positive MRF vs. positive CMR and extended surgery to achieve negative CRM in MRF-infiltrating tumors has been discussed previously (22).

Our finding is consistent with the report of Pettersson et al., who described a significant decrease in CRM-positive cases of 50% at initial staging vs. 14% at pathologic examination (13). For CRT, Bahadoer et al. reported a decrease to 9% CRM-positive cases on pathology in a high-risk population with 30% cT4 tumors and 60% CRM-positive cases at initial staging (9).

When evaluating tumor downsizing based on re-staging data, a partial response is defined as regression of the tumor by at least 30% according to RECIST criteria (23). For SRT delay, there is only one study reporting on tumor downsizing at re-staging. Pettersson et al. described tumor regression in 74% of patients on re-staging MRI, but without quantifying the extent (13).

We found a significant reduction in tumor size at restaging after neoadjuvant radiotherapy in our patients, which translated into a mean reduction in craniocaudal tumor diameter of 29% and tumor volume of 51%. Accordingly, endoscopic re-staging described significant regression (25%–50%) in 36% of patients and extensive regression (>75%) in another 23% of patients. The mean reduction in tumor diameter from initial staging to pathologic examination was more pronounced (51%) than the decrease according to restaging data (29%).

This fact may be caused by both overstaging on MRI due to fibrotic thickening or edema (21) and shrinkage of specimens after formalin fixation (24).

With CRT, Yu et al. reported a mean reduction in craniocaudal tumor length of 33% after MRI re-staging, which is similar to our results after SRT-delay (20).

Furthermore, Yu et al. demonstrated that patients with >50% tumor reduction after CRT showed a survival benefit in addition to the intended improvement in local tumor control. Tumor downsizing of this extent was seen in about 24% of patients in their study (20). In our study, only 14% of patients had a tumor reduction >50% as determined by tumor diameter at MRI re-staging.

On the other hand, no tumor response to SRT-delay was observed in only one patient (5%) in our study, which manifested as pathological regression Dworak grade 0.

In this context, Petterson et al. reported upstaging in 11% of patients after SRT-delay, comparing initial staging with pathologic stage (13). However, further related data based on pathological tumor regression are not available for SRT-delay.

The assessment of response to treatment of solid tumors according to the RECIST criteria focuses on the unidimensional evaluation of the longest tumor diameter (23).

With the increasing availability of novel radiological segmentation software, semi-automated tumor volumetry is a potentially useful additional assessment tool for better detection of tumor response that has been used in several solid tumors (25, 26).

In rectal cancer, measurement of total tumor volume has been shown to be more accurate than measurement of one- and three-dimensional size in assessing response to neoadjuvant treatment (27).

In our study, semiautomated volume measurement documented a mean 51% reduction in tumor volume after neoadjuvant radiotherapy, which is more pronounced than the reduction observed with unidimensional assessment of tumor diameter.

To date, there are no volumetric data on response to SRT-delay on which to benchmark our results. For CRT, Martens et al. reported a mean 65% reduction in total volume and a mean 36% reduction in tumor length (27) (Table 2).

**Table 2:** Downsizing of rectal cancer following neoadjuvant therapy evaluated in restaging MRI. Reduction of tumor diameter, Regression of total tumor volume. CRT, conventional chemoradiation; SRT-delay, short-term radiotherapy with delayed surgery; n.d., not done.

Author	Neoadjuvant regimen	Tumor diameter	Tumor volume
Yu et al. (20)	CRT	−33%	n. d.
Martens et al. (27)	CRT	−36%	−65%
present study	SRT-delay	−29%	−51%

Downstaging, as determined by comparing the initial staging with the pathologic stage, was observed in 68% of patients in our study, although none showed a complete response.

With an interval of 4–5 weeks to surgery after neoadjuvant radiotherapy, Pach et al. reported downstaging in 44% of patients and complete response in 10% (14). The difference in complete response is presumably related to the number of patients in our study.

However, complete response is observed more frequently with CRT, in 12%–20% of patients (7, 9, 10, 20, 27).

Regarding the goal of neoadjuvant treatment to increase the frequency of sphincter-preserving surgery by tumor downsizing, our study cannot provide data as the majority of tumors in our cohort did not have a critical distance to the anal verge. Pach et al. reported no improvement in the rate of sphincter preservation at 4–5 weeks after neoadjuvant radiotherapy with a 2 cm rule for the distal margin (13). In contrast, an increase in sphincter-preserving surgery was noted in up to 25% of patients after CRT (28).

Our study has several limitations, notably the retrospective design, the number of patients included, and the range of the time interval before surgery.

Because of these limitations, the results should be interpreted with caution.

## Conclusion

The present study demonstrated that SRT-delay can lead to significant downstaging and downsizing of locally advanced rectal cancer. The observed extent of downsizing is broadly comparable to the results of CRT, making SRT-delay a serious alternative for patients who cannot tolerate chemotherapy. In our study, semiautomated measurement of total tumor volume was a feasible and accurate tool for assessing downsizing after SRT-delay.

The extent to which SRT-delay in very low rectal cancer may increase the number of sphincter-preserving procedures needs further investigation in an appropriate cohort and design. Also, to investigate whether the extent of downsizing after SRT-delay results in a survival benefit comparable to that of CRT.

## Data Availability

The original contributions presented in the study are included in the article/Supplementary Material, further inquiries can be directed to the corresponding author/s.
